# Comparison between general anesthesia and superficial cervical plexus block in partial thyroidectomies

**DOI:** 10.1016/S1808-8694(15)30758-8

**Published:** 2015-10-19

**Authors:** Rui Celso Martins Mamede, Helder Raful

**Affiliations:** 1 Associate Professor – Department of Ophthalmology, Otorhinolaryngology and Head and Neck Surgery (Head of the Head and Neck Surgery Department - HCFMRP-USP); 2 MD. Graduate Student - Department of Ophthalmology, Otorhinolaryngology and Head and Neck Surgery (Head of the Head and Neck Surgery Department - HCFMRP-USP). Hospital das Clínicas da Faculdade de Medicina de Ribeirão Preto - USP

**Keywords:** ambulatory surgery, anesthesia, thyroidectomy

## Abstract

Thyroidectomy under the effect of superficial cervical plexus block (SCPB) has met resistance.

**Aim:**

to compare variables in patients submitted to hemithyroidectomy under the effect of general anesthesia (GA) and SCPB.

**Case report and Methods:**

GA was used in 21 patients, and SCPB was used in another 21 patients. Following sedation, marcaine 0.5% with vasoconstrictor was used in the SCPB group. Intraoperative sedation with diazepam and metoprolol to control arterial pressure and cardiac frequency was given as needed. GA followed the standard method in the unit.

**Results:**

We found significant results (p<0.05, Student's t-test) for surgery time (GA – 111.4 min; SCPB – 125.5 min), anesthesia time (GA – 154.1 min; SCPB – 488.6 min), time in the surgery room (GA – 15 min; SCPB – 1 min), treatment costs (GA - R$203.2; SCPB - R$87.4), presence of bradycardia (GA – 0; SCPB – 23.8%) and laryngotracheal injury (GA – 51; SCPB – 0 %). We also found the following non-significant results: hospitalization time (GA – 17.3; SCPB – 15.1 hours); bleeding volume (GA – 41,9 g; SCPB – 47.6 g), size of the operative specimen (GA – 52.1 cm3; SCPB – 93.69 cm3) and patient satisfaction level (GA – 3.8; SCPB – 3.9).

**Conclusion:**

Although the incidence of bradycardia was higher (23.8%), SCPB was done for the resection of tumors measuring up to 348 cm3, at a lower cost and with no laryngotracheal injuries; these were present in 51% of patients undergoing GA.

## INTRODUCTION

There is no consensus about the ideal method for anesthesia to reduce the risk of injury or death.^1^^,^^2^ Surgeons prefer general anesthesia, as patients sleep throughout the procedure. Galbeman et al.^3^ compared surgeries done under general anesthesia and superficial cervical plexus block, and noted that there seemed to be advantages when using local anesthesia.

Currently, having learnt the surgical technique that reduces to a minimum the risk associated with thyroidectomy, researchers have tried to establish which is the ideal anesthesia technique for that specific operation; in other words the safest, most effective, best quality (painless), lowest cost, and complication-free technique. Studies have attempted this comparison, but there are significant disagreements.

## OBJECTIVES

The aim of this study was to compare surgical and anesthetic parameters, the cost of treatment, and the degree of satisfaction in hemithyroidectomy patients undergoing two anesthetic techniques (general anesthesia, and superficial cervical plexus block).

## SERIES AND METHOD

This study included 42 patients who underwent hemithyroidectomy performed by the same surgeon; 21 patients were operated under general anesthesia (GA) and 21 patients were operated under superficial cervical plexus block (SCPB), according to the order by which they were admitted to the hospital (Santa Casa de Marilia). The Research Ethics Committee approved the study (protocol number 242/2002).

Exclusion criteria were: patients with an indication for a total thyroidectomy; patients with tumors larger than the cervical segment; those classified above ASA II; patients allergic to the drugs that were used in this study, and those with communication difficulties (deaf and hard of hearing).

The variables that were analyzed were: sex, age (years), weight (kg), height (m), duration of surgery and of anesthesia, duration of hospital stay, time spent in the operating room and in the recovery room, intra- and extraoperatory bleeding, extent of resection, complications, patient satisfaction, number of days with drainage tubes, the need for immediate postoperative analgesia, vital signs (arterial pressure, heart rate) and oxymetry.

The duration of anesthesia for the SCPB group was measured from the moment in which the anesthetic agent was injected into Erb's point until skin sensitivity started to return. Recovery was detected by gently rubbing the skin of the neck with a cotton wad. The initial time for patients undergoing GA was the moment that drugs were injected for the induction of anesthesia, and the end time was when the endotracheal cannula was removed.

The duration of surgery for both groups was measured from the moment the skin was incised until the moment when the dressing had finished being applied.

The criterion for discharging GA group patients from the surgical room was the minimum alveolar concentration of anesthetics, established at between 0.15 and 0.5. The criterion for discharging SCPB group patients from the surgical room - and who were, therefore, sedated - was the Aldrete and Kroulik scale. The criteria for discharging both groups of patients from the hospital were autonomy for feeding and walking.

Intraoperative bleeding was assessed by weighing surgical gauze and sponges used during surgery. Suctioning was not used during these procedures.

Treatment cost analysis took into account the daily hospital fee (R$ 83.00) multiplied by a percentage that corresponded to the number of hours that the patient remained in each hospital sector, to which was added the cost of drugs used during that period. The cost of drugs was based on values quoted in the Brasindice Pharmaceutic Guide – 2005 (Guia Farmacêutico Brasíndice – 2005); for calculation purposes, the value was proportional to the amount used.

Laryngotracheoscopy was done on the first postoperative day to check for the presence of laryngotracheal injury resulting from introducing the laryngoscope or the anesthesia probe.

All of the patients returned for daily postoperative visits at the outpatient unit to evaluate the surgical wound and to annotate the volume of secretions passing through a Porto-Vac 3.2 mm drainage tube placed through an opening in the surgical wound. The tube was removed when the volume was equal to or lower than 5 ml.

Patient satisfaction was quantified based on a 1 to 4 score for the surgical procedure, and on being willing to undergo a new operation under the same type of anesthesia.

Endovenous dipyrone was used for postoperative analgesia, as requested by the patient; this was annotated in the chart to measure pain intensity.

Patients ingested one diazepam 10 mg tablet one hour before surgery.

GROUP I - SUPERFICIAL CERVICAL PLEXUS BLOCK (SCPB): One ampule of meperidine and 50 mg of promethazine were injected intramuscularly for sedation, following venoclysis in the cubital fossa of one arm. Seven to nine milliliters of bupivacaine 0.5% with vasoconstrictor were injected on each side of the neck. Care was taken not to give more than 1 to 2 mg/kg, which would be a toxic dose. The anesthetic was injected immediately below the superficial cervical fascia, along the posterior edge of the sternocleidomastoid muscle, just over its midpoint, above the point of intersection with the superficial jugular vein (Erb's point). Before starting the operation, 10 to 15 ml of lidocaine 2% with adrenalin were injected subcutaneously along the intended incision area and into deep layers (peri-glandular layers) to reduce bleeding. Supplemental anesthesia was given if needed. The maximum dose of lidocaine given was 5 to 7 mg/kg, again to avoid a toxic dose. If needed, 1 mg of metoprolol was used as an endovenous bolus every five minutes to control tachycardia and/or high blood pressure. Sedation was attained with endovenous diazepam 2 mg.

An oxygen catheter was used perioperatively at 2 liters/minute to improve inhaled air and to reach adequate oxymetry (> 90%), which was monitored by a digital oxymeter. The anesthetic team monitored vital signs and gave sedation and beta-blockers.

One endovenous 10 ml ampule of bromopride mixed with 10 ml of glucose 25% was given preoperatively to neutralize the emetogenic effect of meperidine and to avoid hypoglukemia crises during surgery.

GROUP II - GENERAL ANESTHESIA (GA): Patients underwent general anesthesia with orotracheal intubation by anesthetists according to the routine procedures for anesthesia of the Santa Casa de Misericordia de Marilia hospital.

The following substances were used: air, oxygen, nitrous oxide, etomidate, enflurane, fentanyl, pancuronium, droperidol, prostigmine and atropine.

**Statistical analysis**

Student's t test for independent groups was used; the F test and Smith's method were used for testing the homogeneity of variances. Fisher's exact test was used for studying associations.

The adopted level of significance was 5% probability for rejecting the null hypothesis.

## RESULTS

Results were not significant when comparing both groups according to age, body weight and height. Table 1 shows that differences in hospital stay and daily hospital fees were not statistically significant; however, significance was found when comparing the cost of drugs and of the treatment, both of which were higher in GA.

**Table 1:** Distribution of treatment costs (daily hospital fee, drugs and the total) and of hospital stay as related to the type of anesthesia (general anesthesia or plexus block). Statistically significant data (p<0.05) in Student's t test are marked*.

Group Variable	Block (n=21)	General Anesthesia (n=21)	
Hospital Stay (hours)	15,1 +/– 5,3	17,3 +/– 5,9	
Hospital daily fee (R$)	52,7 +/– 18,1	59,4 +/– 19,6	
Cost of Drugs (R$)	34,7 +/– 7,7	143,9 +/– 27,7	*
Cost of Treatment (R$)	87,4 +/– 19,4	203,2 +/– 32,7	*

The difference in the mean duration of surgery was statistically significant; there was an added 14.1 minutes in operating time for SCPB patients (125.5 min) compared to GA patients (111.4 min). The mean duration of anesthesia was significantly higher when using SCPB (488.6 min) compared to GA (154.1 min).

There was no statistically significant difference in the number of days before removing drains (GA – 3.2 days; SCPB – 3.1 days), in loss of blood (GA – 41.9 g; SCPB – 47.6 g), and in the size of the excised specimen (GA – 52.1 cm3; SCPB – 93.69 cm^3^).

Table 2 shows hemodynamic data, revealing that there were no statistically significant differences in mean blood pressure and heart rates.

**Table 2:** shows hemodynamic data, revealing that there were no statistically significant differences in mean blood pressure and heart rates.

Group Variable	Block (n=21)	General Anesthesia (n=21)
Sp max. (mmHg)	148,6 +/– 34,4	137,1 +/– 21,7
Dp max. (mmHg)	88,8 +/– 22,6	85,2 +/– 6,8
Sp min. (mmhg)	113,8 +/– 19,1	104,3 +/– 16,0
HR ent. (bpm)	80,8 +/– 17,2	81,3 +/– 15,6
HR min. (bpm)	70,6 +/– 13,5	74,3 +/– 9,7
HR max. (bpm)	97,4 +/– 18,0	91,3 +/– 13,4
SPD (bpm)	33,0 +/– 24,9	32,9 +/– 20,3

There were no statistically significant differences in the presence or not of complications between both groups; there were 49 complications in the GA group and 57 complications in the SCPB group. Complications that had statistically significant differences were: bradycardia, mostly in SCPB patients, and laryngotracheal trauma in GA patients (Table 3).

**Table 3:** Distribution of perioperative complications as related to the type of anesthesia (general anesthesia or plexus block). The following were noted: oxymetry; maximum systolic pressure (SP max) and maximum diastolic pressure (DP max), minimum systolic pressure (SP min); Heart rate (HR); increased intratracheal pressure; vomiting, dysphagia, agitation, back pain, coughupon dissecting the isthmus, hoarseness, dyspnea and laryngotracheal trauma. Statistically significant data (TEF < 0.05) is marked*.

Group Variable	General Anesthesia	Superficial cervical plexus block
Oximetry < 90 mmHg	–	1
SP min < 90 mmHg	3	1
SP max > 140 mmHg	10	13
DP max > 90 mmHg	11	11
HR > 100 bpm	5	12
HR < 60 bpm	–	5*
Increased intra-trach. pr.	1	–
Hoarseness	1	2
Vomiting	6	3
Dysphagia	1	–
Agitation	0	2
Back pain	–	2
Cough upon dissecting the isthmus	–	4
Dyspnea	–	1
Laryngotracheal trauma	11	–*
TOTAL	49	57

There were no differences in the proportion of satisfied patients operated under GA or SCPB. The mean levels of satisfaction (scored from 1–4) were: 3.8 for GA and 3.9 for SCPB. The proportion of patients that would operate again under the same anesthesia was not statistically different between the GA and SCPB groups. The number of patients that took dipyrone postoperatively was similar in the GA and the SCPB groups (16 in each group).

## DISCUSSION

The sample population included 42 patients of both sexes, of which 38 were female, a female-to-male proportion of 9.5:1. Age ranged from 16 to 72 years, mean 47.5 years. These patients underwent hemithyroidectomy for a variety of conditions that were equally distributed between both groups.

The cost of daily hospital fees was linked to the number of hospital-stay hours, which was similar in both groups (GA – 17.3 hours; SCPB – 15.1 hours). Thus, the mean value of the daily hospital fee in the GA group was R$ 59.40, which was not statistically different from that of the SCPB group (R$ 52.70). Certain measures, such as external medical assessments, patient hospital admission on the same day of surgery, and well-defined postoperative protocols, are measures that surely reduce cost by reducing hospital stay. Other authors, such as Prasad and Shanmugam^4^, also agree that cost is reduced when hospital stay is shorter. These authors believe, however, that this time period is linked to the type of anesthesia, contending that SCPB-anesthetized patients start feeding and walking earlier than patients undergoing the same operation under GA; this was not observed in our patients.

There was a statistically significant difference in the cost of drugs; the mean cost was 4.1 times higher in the GA group (R$ 143.90) compared to the SCPB group (R$ 34.70). Therefore, the total cost of treatment was R$ 203.20 for GA and R$ 87.40 for SCPB.

There was a statistically significant difference in the duration of hemithyroidectomy under GA (111.4 minutes) and under SCPB (125.5 minutes). We agree with Chaikof et al.^5^ who state that technique and ability, added to anatomical knowledge, define the duration of surgery. These authors further state that success is attained when practice and order are added to the aforementioned requirements. The duration of surgery was shorter in patients operated under GA (85 minutes), compared to patients operated under SCPB (175 minutes). Permanence time in the surgical room was similar for both groups; although surgery took longer in the SCPB group, reversion from anesthesia required more time in the GA group. Permanence in the surgical theater was not significantly different (GA – 17.3 hours; SCPB – 15.1 hours. The mean size of the surgical specimen was not statistically different between both groups. The largest specimen was removed during a procedure done under SCPB; it measured 348 cm^3^.

The end of anesthesia is hard to establish in SCPB and in GA. Recovery of cervical skin sensitivity is slow, therefore we decided to detect the moment that sensitivity started to return. We considered the end of GA as the moment the endotracheal tube was removed; however, this does not mean that patients were still not under the effect of anesthesia.

Dieudonne et al.,^6^ Aunac et al.^7^ and Lubenow et al.^8^ have stated that cervical segment anesthesia reduces the need for postoperative dipyrone, which indicates lower postoperative stress. In our study, however, this alleged superiority of SCPB over GA was not confirmed, as the same number of patients requested analgesics. Herbland et al.^9^ reached a similar result when performing SCPB with ropivacaine 0.75%, before or after thyroidectomy in patients under GA; these authors found that the block did not reduce the need for postoperative analgesics.

In our study we found that the mean perioperative maximum systolic arterial blood pressure was similar in both groups, and remained close to the upper normal limit (140 mmHg). Ten patients in the GA group and 13 patients in the SCPB group had high blood pressure peaks (over 140 mmHg). Of these, two in the GA group and three in the SCPB group had a hypertensive peak equal to or over 180 mmHg; the patient presenting the highest blood pressure peak (250 mmHg) was under SCPB. Two GA patients had an arterial blood pressure peak of 180 mmHg (they were controlled high blood pressure patients) that occurred just before surgery; they progressed to normal blood pressure levels during the operation. The blood pressure peaks were probably caused by the release of adrenergic substances due to preoperative stress, as these two patients had normal blood pressure upon hospital admittance. This is a known occurrence, as hypertensive patients present sympathetic hyperactivity when subjected to negative stimuli. Two of the three patients in the SCPB group that had blood pressure peaks equal to or over 180 mmHg required a beta-blocker (metoprolol) and sedation (diazepam) to control their blood pressure; blood pressure in the third patient was controlled by increasing sedation (diazepam). The patient that had the highest blood pressure peak (250 mmHg) was hypertensive throughout the operation (>140 and <180 mmHg), even though significant sedation (diazepam) and a higher dose of a beta-blocker (metoprolol) were used. No patient presented cardiovascular complications. In this context we found no differences between both types of anesthesia, although Galbeman et al.^3^ have reported that perioperative pressure levels were higher when using the SCPB compared to GA.

The mean maximum diastolic blood pressure was similar in both anesthesia techniques: 85.2 mmHg in the GA group and 88.8 mmHg in the SCPB group, both within normal limits. Twenty-two patients, 11 of whom in the GA group, had diastolic blood pressure peaks over 90 mmHg, but never above 110 mmHg. The data in our sample allow us to state that both anesthesia groups carried a similar cardiac risk.

A comparison of the mean minimum systolic blood pressure between groups according to the t test yielded a non-significant result (p>0.05), allowing us to join Mangano10 in stating that both anesthesia groups have the same possibility of presenting hypotensive systolic peaks. These peaks occurred in three GA group patients and in one SCPB group patient. Pressure in these four patients fell to 80 mmHg with no apparent cause, and progressed uneventfully.

According to our study, both GA and SCPB patients had the same perioperative mean pressure variation (SPD = systolic pressure difference), in other words, they had similar perioperative cardiac morbidity; we, therefore, are unable to state that the SCPB is safer than GA.

The heart rate upon entry into the surgical room, and the minimum and maximum heart rates, were statistically similar in both anesthesia groups. The mean maximum heart rate did not exceed 100 bpm, remaining at 91.3 bpm in the GA group and 97.4 bpm in the SCPB group. There were no complications that could be attributed to tachycardia in both groups. According to Mangano10 and Braz,11 keeping an adequate heart rate decreases myocardial oxygen consumption, thereby reducing the risk of infarction. In our study we successfully used beta-blockers as our perioperative tachycardia-correcting strategy.

We do not consider tachycardia as a complication; its presence was not associated with the intravascular injection of anesthetics, for two reasons: tachycardia did not coincide with the moment anesthetics were injected, and secondly, we followed the technical principle of aspirating for blood to be sure the needle was not within a blood vessel, as defended by Harris and Benveniste,^12^ and Rodrigues Jr. et al.^13^

One of the measures taken to avoid toxicity was to use anesthetics with vasoconstrictors. This association minimizes toxicity by reducing plasmatic levels of these drugs.

Patients had a similar mean volume of perioperatory blood loss; bleeding in the GA group was 5 to 167 grams (mean – 41.9 grams), and bleeding in the SCPB group was 5 to 146 grams (mean – 46.7 grams). A similar situation occurred with the volume of blood drained through the drainage tubes; these tubes remained in GA group patients for 3.2 days and in SCPB group patients for 3.1 days. We believe that the surgical technique, the type of instrumentation, the tumor type and volume, the presence of systemic disease, and knowledge of the cervical anatomy explain increased or decreased bleeding, and is independent of the technique used for anesthesia.

Laryngobronchoscopy was done on the first postoperative day in GA group patients; we found traumatic injury in 11 of 21 patients belonging to this group (51%). Although these injuries were mild, they may prefigure severe lesions, such as stenosis.

One of the causes of anesthetic conversion is the inability to maintain adequate oxygenation of the blood, which affects the quality and efficacy of anesthesia. One patient, who belonged to the SCPB group, had an oxymetry reading below 90% (87%). This episode lasted about 10 minutes and was reverted by increasing the amount of oxygen, after which surgery proceeded as usual. We believe that sedation is another factor that affects oxymetry; we thus agree with Prasad and Shanmugam^4^ in stating that comprehensive anesthesia involving sedation is a mixture of art and science in which the contribution of each is hard to measure, and which depends on individual involvement. Added to this is individual susceptibility, as patients with similar traits responded differently to the similar doses of the same sedative. We, therefore, believe that there is no formula for keeping all of the patients under a common sedation level.

We found that there were no statistically significant differences between both groups in the level of patient satisfaction. Most of the patients (18 from each group) reported themselves as being extremely pleased, having given the maximum score to this question. One GA group patient gave score 2, explaining that he had felt significant pain. We agree with Chaikof et al.,^5^ Lo Gerfo,^14^ and Prasad and Shanmugam4 in stating that to perform SCPB, anatomical knowledge together with technical expertise will result in a pain-free operation and a positive experience for the patient.

Most of the patients (20 of 21 in the GA group, and 19 of 21 in the SCPB group) would accept hemithyroidectomy again under the same type of anesthesia. The only GA group patient that reported he would not undergo GA again explained that he had felt significant immediate postoperative pain and a disagreeable feeling during the induction of anesthesia. The two SCPB group patients that would not accept this method a second time alleged that they were very tense, and that they believed GA would provide superior relaxation and complete amnesia (also mentioned by Prasad and Shanmugam^4^).

Given these results, we agree with Prasad and Shanmugam,^4^ and Telford, Stoneham and Pandit^15^ in defending SCPB, as this method can induce anesthesia at a lower cost with fewer complications that, when present, are of lower risk; this type of anesthesia also provides adequate patient satisfaction. We also agree with Chaikof et al.^5^ that an association between anesthesia and surgical technique is essential for major interventions. It is possible that SCPB might be used even in major procedures, such as laryngectomy and cervical dissection, as long as the Prasad e Shanmugam^4^ concepts are added, which state that detailed anatomical knowledge of the neck increases the effectiveness of anesthesia. This results in adequate muscle relaxation that, when associated with sedation and gentle handling of surgical tools and organic tissues, makes it possible to operate deeper anatomical levels. Chaikof et al.^5^ state that practice should be associated with order for such procedures. We believe, however, that further studies with larger samples and in different geographies and cultures are needed.

## CONCLUSION

The results led to the following conclusions:

– the duration of surgery was increased when using the SCPB;
– the time for reversion in the surgical room was increased when using GA;
– the duration of anesthesia was increased when using SCPB and did not imply in less use of analgesics during the immediate postoperative period;
– there was laryngotracheal trauma in 51% of patients undergoing GA, and bradycardia in 23.8% of the patients undergoing SCPB;
– treatment costs were lower when using SCPB.
